# Development and validation of an interpretable machine learning model for pulmonary heart disease in patients with pneumoconiosis

**DOI:** 10.3389/fpubh.2026.1952855

**Published:** 2026-09-23

**Authors:** Yifei Xie, Yi Feng, Xinglin Wan, Haoyu Mao, Lijun Peng, Wen Du, Dingzi Zhou, Yuqin Yao, Qin Zhang, Shangcheng Xu

**Affiliations:** 1 West China School of Public Health and West China Fourth Hospital, Sichuan University, Chengdu, China; 2 Chongqing Key Laboratory of Prevention and Treatment for Occupational Diseases and Poisoning, Chongqing Prevention and Treatment Center for Occupational Diseases, Chongqing, China; 3 Occupational Health and Occupational Medicine Key Laboratory of Sichuan Provincial Health Commission, Chengdu, China; 4 Molecular Toxicology Key Laboratory of Sichuan Provincial Education Office, West China School of Public Health and West China Fourth Hospital, Sichuan University, Chengdu, China

**Keywords:** complications, machine learning, pneumoconiosis, pulmonary heart disease, risk screening

## Abstract

**Background:**

Pneumoconiosis patients with pulmonary heart disease (PHD) face an elevated risk of mortality. Early identification of individuals at higher risk of PHD is important for timely risk stratification and clinical management. However, few risk-screening models have been specifically developed for pneumoconiosis in primary care settings, where access to advanced diagnostic resources may be limited. Therefore, this study developed and validated a machine learning model based on routinely available data to support early risk stratification and clinical decision-making in resource-limited environments.

**Method:**

A dual-design occupational pneumoconiosis cohort, comprising retrospective and prospective components, was established using occupational, epidemiological, and laboratory data obtained from electronic medical records and questionnaires. Key variables were selected using least absolute shrinkage and selection operator (LASSO) regression; five machine learning models were developed using five-fold cross-validation with grid search for hyperparameter tuning. Model performance was evaluated using the area under the receiver operating characteristic curve (AUC), F1 score, accuracy, precision-recall area under the curve (PR AUC), calibration metrics, and threshold analysis. The prospective cohort was used to evaluate the generalizability of the models. Model interpretability was assessed by Shapley additive explanations (SHAP).

**Results:**

Following strict application of the inclusion and exclusion criteria, the retrospective cohort included 984 hospitalized patients (2012–2021), whereas the prospective cohort comprised 829 patients enrolled since 2021. LASSO identified 10 variables for model construction. All models demonstrated satisfactory discrimination, with logistic regression (LR) achieving an AUC of 0.816 in the external validation cohort. Lower BMI, albumin, and platelet large cell ratio (P-LCR), as well as higher red blood cell distribution width coefficient of variation (RDW-CV), older age, and stage III pneumoconiosis, were associated with higher predicted risk of PHD. The SHAP interaction network further indicated prominent statistical interaction patterns among hematological abnormalities, nutritional status, and disease severity, based on their joint contributions to model predictions.

**Conclusion:**

This study indicates that machine learning models, particularly LR, may be useful for PHD risk screening and stratification among patients with pneumoconiosis. SHAP analysis identified clinically accessible variables that were important for model predictions, indicating a multidimensional pattern of associations across hematological, nutritional, and disease-severity domains.

## 1 Introduction

Pneumoconiosis is a chronic occupational lung disease caused by prolonged inhalation and pulmonary retention of production-related dust, characterized by persistent inflammation and irreversible diffuse pulmonary fibrosis (1). As one of the most prevalent occupational diseases globally, pneumoconiosis can cause substantial impairment of lung function and impose a sustained economic burden on affected individuals, families, and society. The Global Burden of Disease (GBD) study indicates that the global burden of pneumoconiosis remains substantial (2). According to the 2019 GBD study, pneumoconiosis accounted for approximately 900,000 disability-adjusted life years (DALYs) lost globally (3). In China, approximately 1.368 million prevalent cases were reported in 2019, accounting for more than 60% of the global burden (4). Although ongoing improvements in occupational disease surveillance and management have contributed to a decline in the reported number of occupational pneumoconiosis cases in China (5), pneumoconiosis remains without a curative treatment. Its chronic progression may result in long-term reductions in work capacity and quality of life, as well as adverse effects on mental well-being, while imposing substantial socioeconomic costs (6). Therefore, pneumoconiosis remains a major priority for occupational disease prevention and control in China (4).

Cardiovascular complications, particularly pulmonary arterial hypertension (PAH) and pulmonary heart disease (PHD), are major contributors to disability and mortality among patients with pneumoconiosis (7). Progressive dust-induced lung injury promotes pulmonary vascular remodeling and elevated pulmonary arterial pressure, increasing right ventricular afterload and ultimately leading to PHD, thereby markedly raising mortality risk (8, 9). Clinical studies indicate that over one-third of pneumoconiosis patients with large shadows develop pulmonary hypertension, resulting in right ventricular overload, dilatation, hypertrophy, and progression to PHD (10). Furthermore, epidemiological studies suggest that long-term silica exposure is associated with an increased risk of cardiovascular disease, especially PHD (11, 12). Therefore, early identification of patients at increased risk of PHD is an important component of pneumoconiosis management and may support timely risk stratification and clinical decision-making.

In recent years, machine learning has advanced rapidly in medicine, particularly in risk screening and prediction for chronic respiratory diseases such as chronic obstructive pulmonary disease (COPD) and pulmonary fibrosis (13). By capturing complex nonlinear relationships and integrating multiple sources of clinical data, machine learning models may provide useful predictive information, while interpretable methods can help characterize the contribution of individual variables to model predictions and support more transparent clinical decision-making (14). However, models specifically developed for PHD risk screening in patients with pneumoconiosis remain scarce. One logistic regression (LR) model incorporating disease duration, pneumoconiosis stage, and the Tei index was developed to assess PHD risk, but its generalizability was limited (15). Another LR model, restricted to silicosis patients, used pulmonary hypertension rather than PHD as the outcome (16). A cohort study identified platelet count and plateletcrit as factors associated with PHD risk but did not develop a risk-prediction tool (17). These studies were conducted at single centers, lacked external validation, and did not employ machine learning approaches beyond conventional LR. Although pulmonary function tests, computed tomography (CT), and echocardiography are well-established approaches for assessing disease severity in pneumoconiosis and PHD, their routine use for population-level risk screening may be constrained by limited accessibility, cost, and the need for specialized personnel and equipment. In primary-care settings, access to these advanced clinical assessments may be limited. Therefore, a practical risk-stratification approach based on routinely available clinical and laboratory data may be useful for early identification of individuals at higher predicted risk of PHD.

Leveraging two independent cohorts from West China Fourth University Hospital, we developed and validated a parsimonious machine learning model using routinely available variables for PHD risk screening and stratification among patients with pneumoconiosis. Designed for potential use in resource-limited settings, the model provides an interpretable approach for estimating predicted risk and may support timely risk stratification and clinical decision-making.

## 2 Methods

### 2.1 Study population

The study population comprised occupational pneumoconiosis patients from retrospective and prospective cohorts at West China Fourth Hospital, Sichuan University. The retrospective cohort included hospitalized patients identified through the hospital information system (HIS) between 2012 and 2021, whereas the prospective cohort included hospitalized and outpatient patients from 2021 onward. All patients were diagnosed and staged in accordance with the Diagnosis of Occupational Pneumoconiosis (GBZ 70–2015) (18). These cohorts were reviewed and approved by the Medical Ethics Committee of West China Fourth Hospital, Sichuan University (Approval No.: HXSY-EC-2021053). This study was conducted and reported following the Transparent Reporting of a Multivariable Prediction Model for Individual Prognosis or Diagnosis (TRIPOD) guidelines for the development and validation of prediction models (19).

This study imposed no sex-based restrictions; however, females accounted for only 1% of the cohort, reflecting the underlying occupational distribution. Because the number of female participants was insufficient for reliable subgroup analysis, female patients were excluded from the final analysis. Inclusion criteria included: (1) diagnosis of occupational pneumoconiosis and staging according to the Diagnostic Criteria for Occupational Pneumoconiosis (GBZ 70–2015) (18); (2) PHD identified through an initial screening of ICD-10 codes (I26–I28 and I27.0–I27.9), followed by manual chart review to confirm the diagnosis according to the Chinese Medical Association’s Guidelines for Primary Care of Chronic Pulmonary Heart Disease (2018) (20). Confirmation required echocardiographic evidence of pulmonary hypertension (sPAP ≥40 mmHg) or right ventricular dysfunction, together with clinical signs of right heart failure and exclusion of other etiologies; (3) Complete clinical data; (4) Informed consent. Exclusion criteria included: (1) Female patients; (2) Unclear stage or diagnosis of pneumoconiosis; (3) More than 30% of clinical data missing; (4) Severe diseases or other comorbidities, including cardiac disease of other etiologies; chronic thromboembolic pulmonary hypertension (CTEPH); active pulmonary tuberculosis or tuberculous pleurisy; severe interstitial or pleural disease attributable to causes other than pneumoconiosis; malignancy; severe hepatic or renal insufficiency at the decompensated stage; or other life-threatening conditions. The primary outcome was the presence of PHD among patients with pneumoconiosis. The case group comprised patients diagnosed with occupational pneumoconiosis who subsequently developed chronic PHD during the observation period. The control group comprised patients with pneumoconiosis but without PHD or other severe complications. Figure 1 illustrates the inclusion and exclusion process for this study.

**Figure 1:**
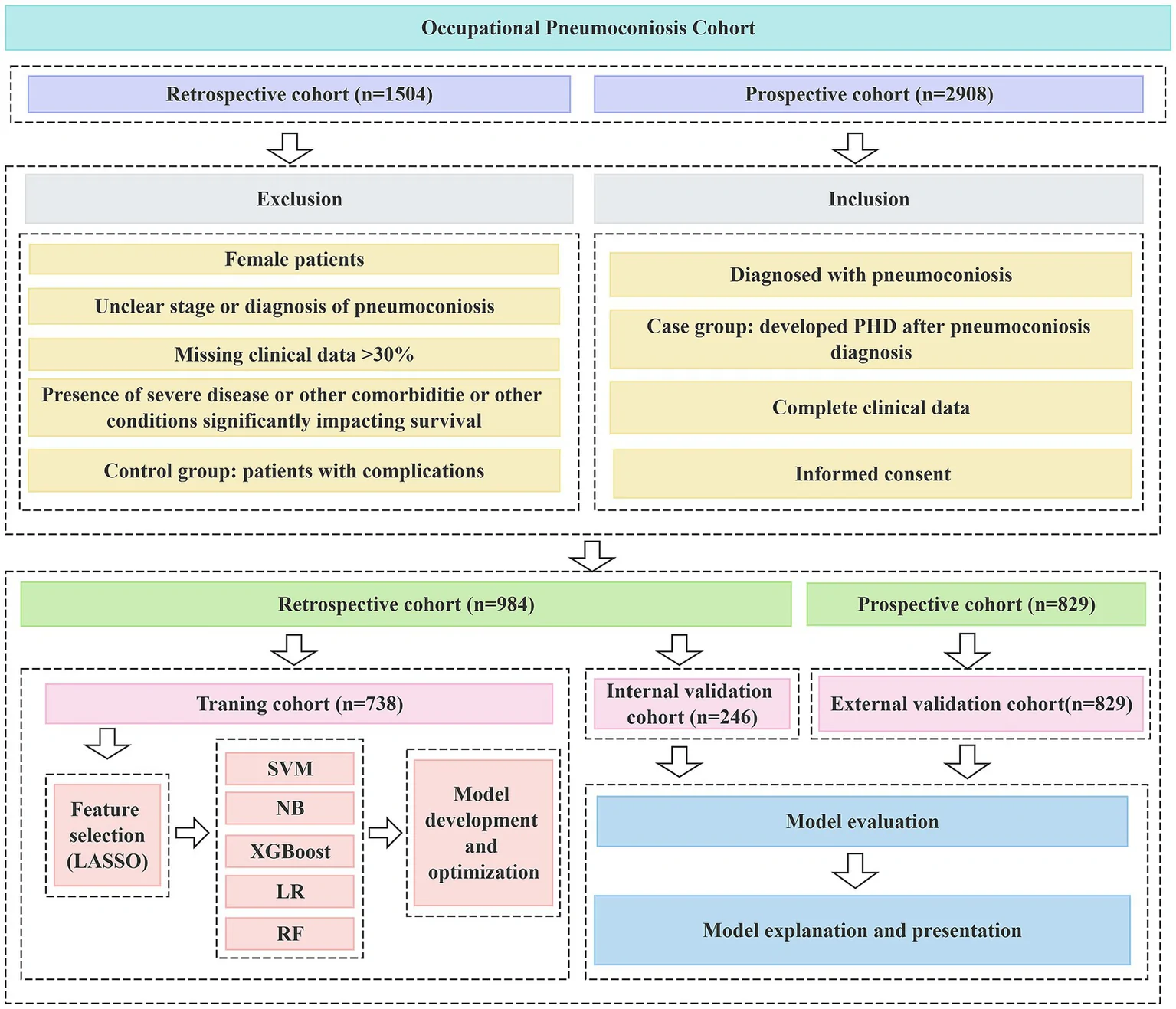
Analysis workflow for the development and evaluation of models.

### 2.2 Datasets development

Demographic, occupational, clinical, and laboratory variables were collected from medical records. A rigorous data preprocessing workflow was implemented to ensure data quality. Duplicate patient records were removed based on unique identification codes. The proportions of missing data were comparable between the retrospective and prospective cohorts, with no variable having more than 10% missingness. Missing values were imputed using the median for continuous variables and the mode for categorical variables. Variables were standardized and encoded: unordered categorical variables were one-hot encoded to avoid imposing spurious numerical order; ordered categorical variables were encoded using an ordinal scheme to preserve their inherent order; and continuous variables were standardized to a mean of 0 and a standard deviation of 1. All preprocessing steps were fitted only on the training cohort and applied to the internal and external validation cohorts using fixed parameters to prevent information leakage. Sample adequacy was assessed based on the events per variable (EPV) principle (21). A total of 21 candidate variables were considered, corresponding to 22 regression parameters after dummy coding. The retrospective cohort included 182 events (EPV = 8.3), while the prospective cohort included 144 events (EPV = 6.5). To reduce the potential for overfitting, the least absolute shrinkage and selection operator (LASSO) (22) was applied for variable selection and shrinkage. Ten variables (11 parameters after expansion) were retained, increasing EPV to 16.5 and 13.1 in the respective cohorts.

### 2.3 Model development

The retrospective cohort was randomly divided into a training cohort and an internal validation cohort at a 3:1 ratio, while the prospective cohort was used for independent external validation. Feature selection was performed in the training cohort using LASSO regression, which facilitated model interpretability by shrinking the coefficients of less informative variables toward zero and thereby selecting a parsimonious set of features. The optimal regularization parameter was determined using grid search with five-fold stratified cross-validation. The 10 features selected by Lasso regression, ranked by coefficient absolute value, were incorporated into the model. Five algorithms, logistic regression (LR), random forest (RF), extreme gradient boosting (XGBoost), support vector machine (SVM), and naïve Bayes (NB), were selected to represent four supervised learning paradigms: parametric(LR), tree-based ensemble (RF for bagging, XGBoost for boosting), kernel-based(SVM), and probabilistic(NB). This selection spans a range of model complexity, enabling systematic evaluation of predictive performance, interpretability, and generalizability while avoiding redundant variants within the same paradigm. All models employed class weight balancing to address class imbalance. A fixed random seed (42) was used throughout the analysis to ensure reproducibility. Five-fold cross-validation randomly divided the training data into five mutually exclusive subsets. Each fold was successively used as the validation set while the remaining four folds served as the training set, repeated five times to achieve a more robust performance evaluation. Grid search was applied for hyperparameter tuning based on cross-validation results within the training cohort.

### 2.4 Model evaluation and interpretation

Receiver operating characteristic (ROC) curves and area under the ROC curve (AUC) were used as primary metrics to evaluate model discrimination in both the internal validation cohort (*n* = 246) and the external validation cohort (*n* = 829). The 95% confidence intervals (CIs) for AUCs were estimated using bootstrap resampling with 1,000 iterations. Additional metrics included precision, recall, F1 score, accuracy, and area under the precision-recall curve (PR AUC). Model calibration was assessed using calibration curves before and after Platt scaling, which was applied to correct systematic miscalibration when the model was evaluated in the external cohort (23). Calibration performance was quantified using the Brier score and Brier Skill Score (BSS), with positive BSS values indicating improvement relative to the specified reference model. Global and individual-level model interpretation was performed using SHAP (Shapley Additive Explanations) (24, 25), including feature importance ranking, individual prediction explanations, feature dependencies, and feature interaction analysis. Given the low prevalence of PHD among patients with pneumoconiosis, clinical decision threshold analysis was performed to assess model performance across a range of decision thresholds. Sensitivity, specificity, positive predictive value (PPV), and number needed to screen (NNS) were calculated at thresholds ranging from 0.05 to 0.50 to characterize the potential clinical utility of the model. These analyses provided threshold-specific information to support the assessment of the model for PHD risk screening in low-prevalence settings.

### 2.5 Statistical analysis

All statistical analyses were performed using Python 3.12. Continuous variables were summarized as the mean ± standard deviation or median (interquartile range), while categorical variables were summarized as frequencies (percentages). Intergroup comparisons employed independent-samples *t*-tests or chi-square tests. A two-sided *p* < 0.05 was considered statistically significant.

## 3 Results

### 3.1 Characteristics of study cohorts

After applying the exclusion criteria, the retrospective cohort comprised 984 patients (182 PHD cases, 18.5%) and was randomly divided in a 3:1 ratio into training cohort (*n* = 738) and an internal validation cohort (*n* = 246). The external validation cohort included 829 patients, of whom 144 had PHD (17.4%). The workflow for cohort construction and selection was illustrated in Figure 1. Baseline characteristics of patients in the training, internal, and external validation cohorts were summarized in Table 1. The internal validation cohort was generally comparable to the training cohort, whereas the external validation cohort showed greater heterogeneity in demographic characteristics, occupational history, and clinical and laboratory parameters. This heterogeneity reflects variability in patient characteristics and clinical settings and provides an opportunity to assess the generalizability of the model. Detailed baseline comparisons for the retrospective cohort were presented in Supplementary Table S1.

**Table 1:** Training cohort, internal validation cohort, and external validation cohort baseline data.

Feature	Training cohort (*n* = 738)	Internal Validation cohort (*n* = 246)	External Validation cohort (*n* = 829)	*p*-value, TVC vs IVC	*p*-value, TVC vs EVC
Age, years	53.5 ± 11.0	53.4 ± 11.2	56.3 ± 9.3	0.901	<0.001
BMI, kg/m^2^	22.2 ± 3.6	22.3 ± 3.8	22.8 ± 3.4	0.964	0.003
Dust exposure time, years	10.5 ± 8.0	11.4 ± 8.6	16.6 ± 10.3	0.231	<0.001
Smoker, n (%)				0.534	<0.001
Yes	632 (85.6)	206 (83.7)	342 (41.3)		
No	106 (14.4)	40 (16.3)	487 (58.7)		
Pneumoconiosis type, n (%)				0.887	<0.001
Silicosis	656 (88.9)	218 (88.6)	637 (76.8)		
Coal worker’s pneumoconiosis	65 (8.8)	21 (8.5)	121 (14.6)		
Other pneumoconioses	17 (2.3)	7 (2.8)	71 (8.6)		
Pneumoconiosis stage, n (%)				0.607	<0.001
Stage I	177 (24.0)	54 (22.0)	276 (33.3)		
Stage II	171 (23.2)	53 (21.5)	189 (22.8)		
Stage III	390 (52.8)	139 (56.5)	364 (43.9)		
TC, mmol/L	4.3 ± 0.9	4.4 ± 0.9	4.5 ± 0.9	0.125	<0.001
LDL-C, mmol/L	2.9 ± 0.8	2.9 ± 0.8	3.2 ± 0.9	0.501	<0.001
HDL-C, mmol/L	1.2 ± 0.3	1.2 ± 0.3	1.2 ± 0.3	0.054	0.086
TG, mmol/L	1.3 ± 0.9	1.3 ± 0.8	1.8 ± 1.5	0.844	<0.001
GLU, mmol/L	5.3 ± 1.7	5.5 ± 1.8	5.8 ± 1.7	0.128	<0.001
UA, μmol/L	341.3 ± 106.0	347.9 ± 102.4	359.3 ± 88.4	0.278	<0.001
ALB, g/L	37.9 ± 5.4	37.9 ± 5.8	43.3 ± 3.8	0.658	<0.001
WBC, ×10^9^/L	7.6 ± 3.5	7.7 ± 3.8	7.0 ± 2.5	0.679	0.004
RBC, ×10^12^/L	4.8 ± 0.6	4.8 ± 0.7	4.9 ± 0.6	0.578	0.975
RDW-SD, fL	44.1 ± 4.7	44.3 ± 4.0	45.0 ± 3.7	0.420	<0.001
RDW-CV, %	13.8 ± 1.4	13.8 ± 1.5	13.6 ± 1.2	0.641	0.001
PDW, %	15.5 ± 2.0	15.6 ± 1.8	16.4 ± 0.4	0.919	<0.001
MPV, fL	10.7 ± 1.6	10.7 ± 1.7	10.8 ± 1.8	0.947	0.930
P-LCR, %	30.9 ± 10.7	31.1 ± 12.1	31.8 ± 12.4	0.977	0.483
PCT, %	0.2 ± 0.1	0.2 ± 0.1	0.2 ± 0.1	0.378	<0.001

### 3.2 LASSO regression for selecting predictive features of PHD in patients with pneumoconiosis

Twenty-one features covering demographic, clinical, and laboratory variables were initially considered. LASSO regression was employed for feature selection, identifying 10 variables with the largest absolute coefficient values: body mass index (BMI), platelet large cell ratio (P-LCR), age, pneumoconiosis stage, albumin (ALB), RDW-CV, blood glucose (GLU), pneumoconiosis type, mean platelet volume (MPV), and smoking status. These selected features represented demographic, disease-related, nutritional, and hematological domains relevant to the model’s predictions.

### 3.3 Performance of the ML model in dual-cohort

The predictive performance of five machine learning algorithms in the internal and external validation cohorts is presented in Figures 2A,B. In the internal validation cohort, AUCs ranged from 0.748 to 0.808, with LR achieving the highest AUC (0.808, 95% CI 0.744–0.867). In the external validation cohort, all models demonstrated good discrimination, with AUCs exceeding 0.750 (LR: 0.816; RF: 0.802; XGBoost: 0.786; SVM: 0.773; NB: 0.755). Table 2 summarizes the performance of five machine learning algorithms in both the internal and external validation cohorts. LR also achieved the highest recall and F1 score in the external validation cohort, indicating a relatively favorable balance between sensitivity and overall classification performance. Calibration curves are shown in Supplementary Figure S1. After recalibration, LR achieved the lowest Brier score (0.1187) and a positive BSS (0.173) in the external validation cohort, indicating improvement relative to the reference model. Brier scores and BSS values before and after recalibration for each model in the internal and external validation cohorts are presented in Supplementary Table S2. Overall, LR demonstrated a favorable combination of discrimination and calibration performance across the validation cohorts. Although the low prevalence of PHD was associated with lower recall and PR AUC, AUC values remained relatively stable, suggesting that model discrimination was less affected by differences in outcome prevalence across cohorts.

**Figure 2:**
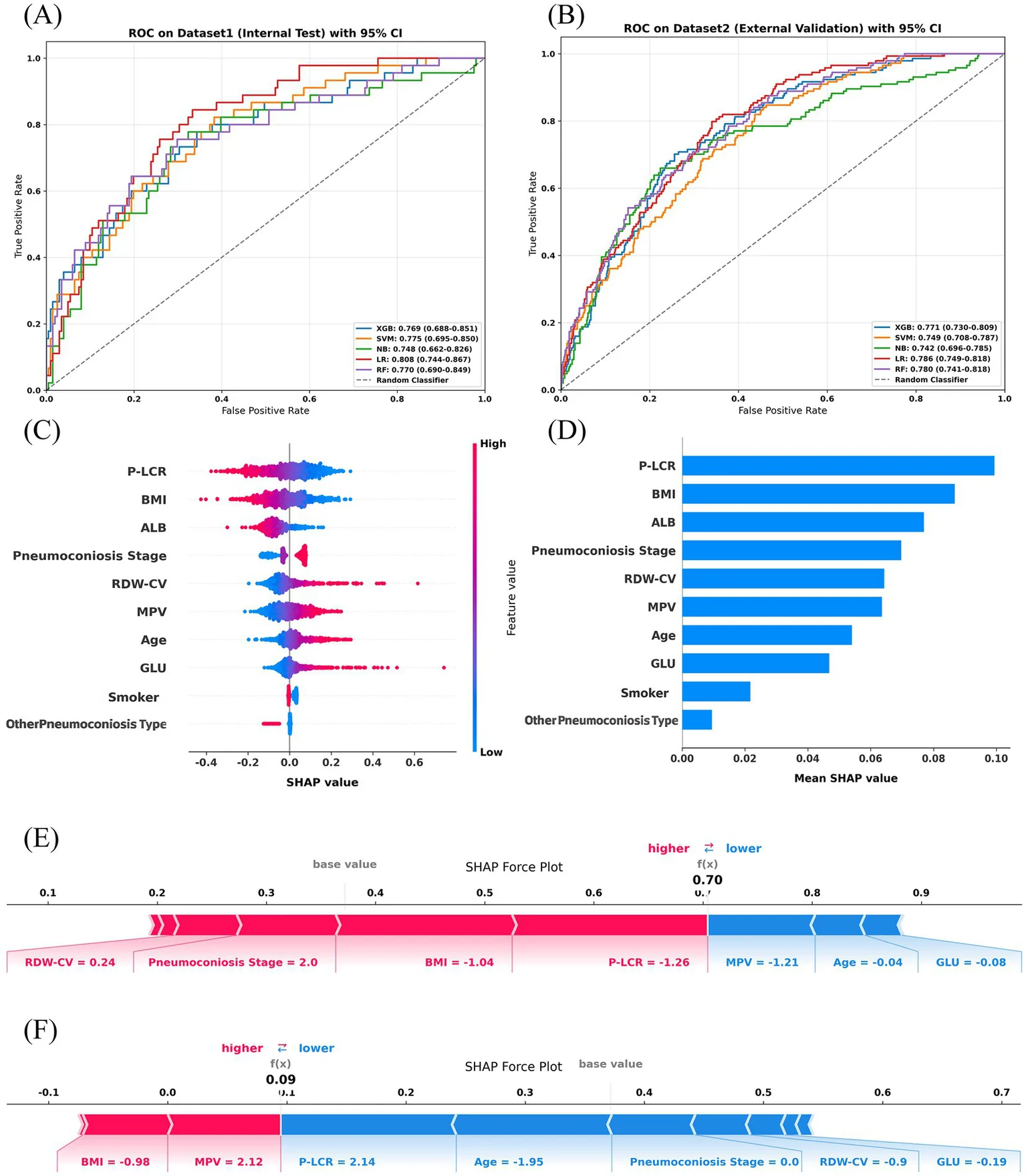
Model performance and interpretability for predicting pulmonary heart disease in pneumoconiosis patients. **(A,B)** ROC curves of five models on the internal validation cohort and external validation cohort. **(C)** A summary plot of the SHAP values for each feature. **(D)** The importance ranking of features based on the mean (|SHAP value|). **(E)** SHAP force plot for pneumoconiosis patients with PHD. **(F)** SHAP force plot for a pneumoconiosis patient without PHD. The higher the SHAP value of the feature, the larger the impact of the feature on the model. The red dots in the feature value represent higher values for that individual patient, whereas the blue dots indicate lower feature values. P-LCR, Platelet Large Cell Ratio; BMI, Body Mass Index; ALB, Albumin; MPV, Mean platelet volume; RDW-CV, Red Blood Cell Distribution Width-Coefficient of Variation; GLU, Blood glucose.

**Table 2:** Performance metrics of different machine learning algorithms in the validation cohort.

Model	CV AUC (train-folds)	Test1 ROC AUC	Test1 PR AUC	Test1 Acc	Test1 Prec	Test1 Recall	Test1 F1	Ext2 ROC AUC	Ext2 PR AUC	Ext2 Acc	Ext2 Prec	Ext2 Recall	Ext2 F1
LR	0.848	0.808	0.471	0.748	0.392	0.689	0.500	0.816	0.482	0.797	0.432	0.528	0.475
NB	0.835	0.770	0.524	0.805	0.467	0.467	0.467	0.802	0.491	0.836	0.561	0.257	0.352
RF	0.834	0.769	0.537	0.797	0.439	0.400	0.419	0.786	0.431	0.820	0.468	0.250	0.326
SVM	0.835	0.775	0.486	0.825	0.542	0.289	0.377	0.773	0.420	0.836	0.625	0.139	0.227
XGB	0.831	0.748	0.405	0.801	0.458	0.489	0.473	0.755	0.386	0.819	0.473	0.361	0.409

### 3.4 Optimal model interpretability

At the global level, the SHAP summary plot illustrates the relative importance of features and the direction of their contributions to model predictions. A positive SHAP value indicates that a feature contributes to a higher predicted probability of PHD, with larger positive values indicating a greater contribution to the model output. The three most influential features were P-LCR (0.096), BMI (0.082), and ALB (0.077), all of which showed predominantly negative contributions to the model predictions (Supplementary Table S3). In contrast, pneumoconiosis stage, RDW-CV, MPV, age, and blood glucose showed predominantly positive contributions to the predicted probability of PHD. In the SHAP summary plot (Figures 2C,D), smoking status showed a relatively weak overall contribution, with SHAP values clustered around zero among smokers and a slight positive shift among non-smokers.

SHAP Force Plots (Figures 2E,F) were used to interpret the model at the individual level. These plots illustrate how patient-specific features contributed to the predicted probability of PHD for individual patients. Representative patients with high and low predicted probabilities were selected for illustration. In Figure 2E, higher RDW-CV, lower P-LCR and BMI, and advanced pneumoconiosis stage showed positive contributions to the predicted probability of PHD, whereas lower blood glucose, younger age, and earlier-stage pneumoconiosis showed negative contributions (Figure 2F). SHAP dependence plots were used to examine the relationships between individual feature values and their contributions to model predictions, illustrating how SHAP values varied across the range of feature values (Figure 3A). For example, lower BMI values and more advanced pneumoconiosis stages were associated with higher predicted PHD risk, while silicosis and coal workers’ pneumoconiosis were associated with higher predicted risk than other pneumoconiosis types.

**Figure 3:**
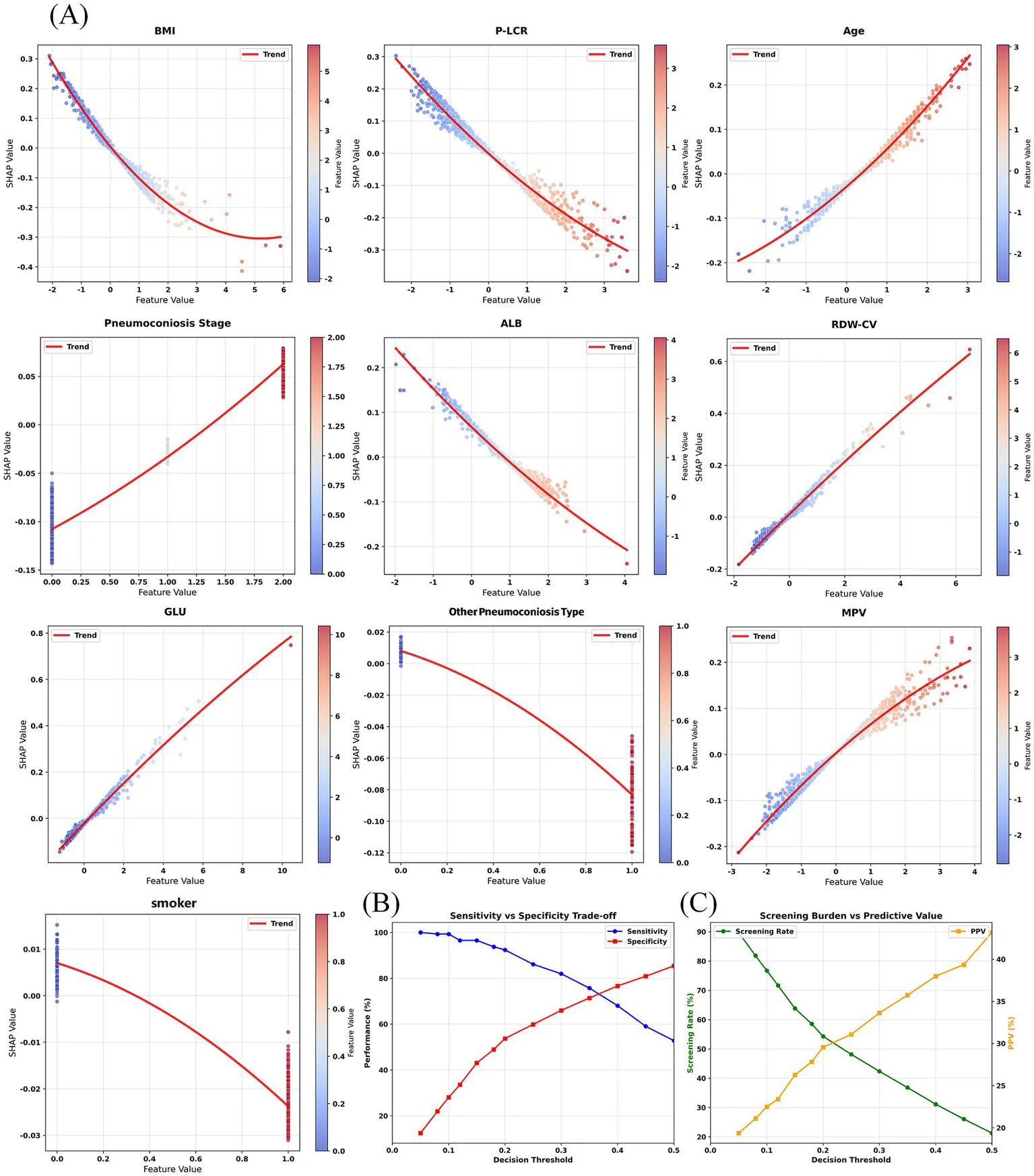
SHAP analysis and model evaluation for predicting PHD risk in pneumoconiosis patients. **(A)** The SHAP dependence plot for features included in the prediction model for the pneumoconiosis patients. The plot shows the relationship between feature values and SHAP values. The y-axis represents the SHAP value of the feature, corresponding to the standardized feature value on the x-axis. **(B)** Sensitivity vs Specificity Trade-off Plot. **(C)** Screening Burden and Predictive Value Plot.

The SHAP variable importance and interaction network plot illustrates the main effects and statistical interaction patterns of the selected features in relation to model predictions (Figure 4). P-LCR and BMI showed the largest main effects and made the greatest contributions to the model predictions. The strongest statistical interaction patterns were observed between P-LCR and BMI, P-LCR and GLU, and pneumoconiosis stage and P-LCR. In contrast, pneumoconiosis type and smoking history showed relatively weak main effects and statistical interaction patterns.

**Figure 4:**
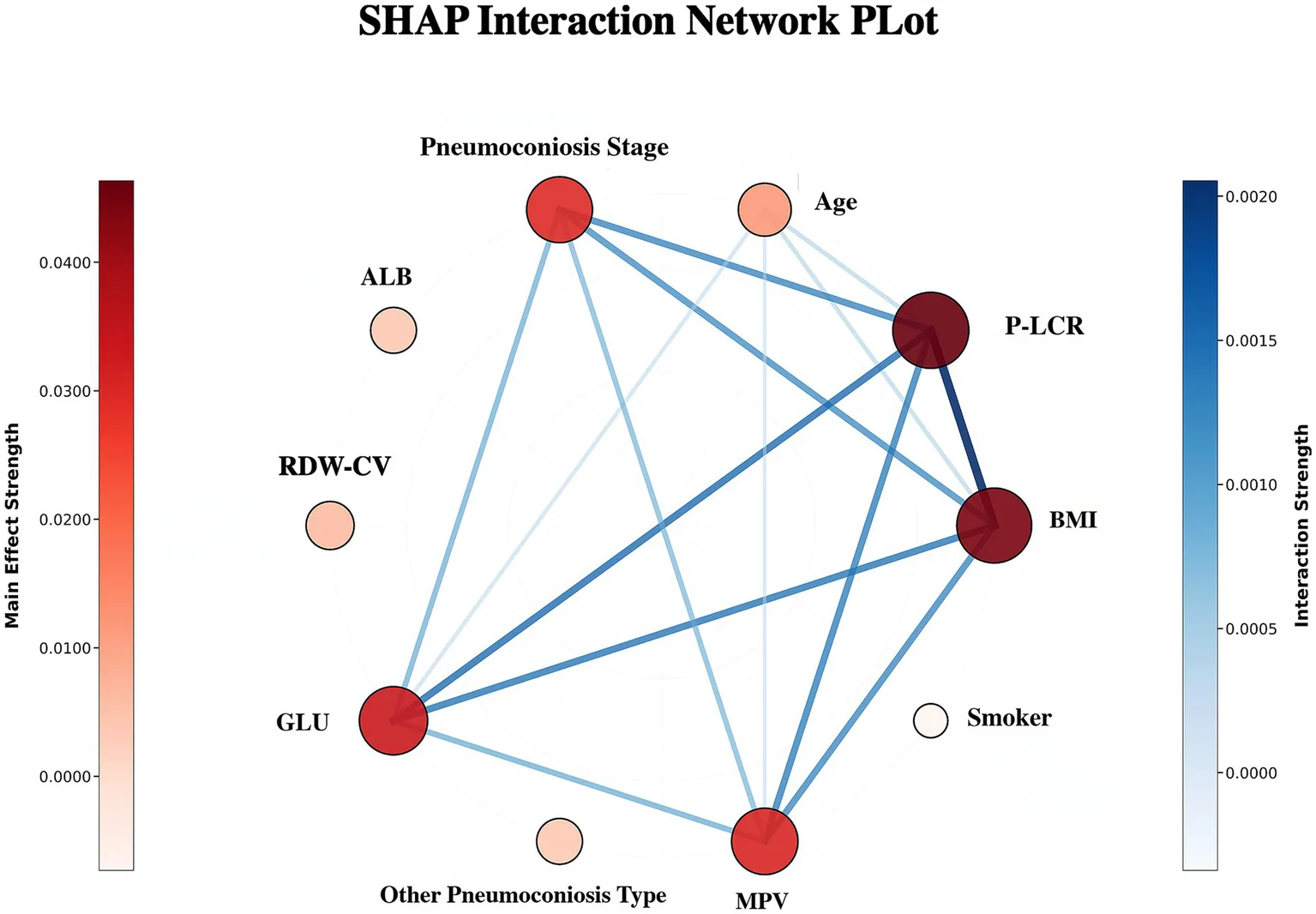
SHAP variable importance and variable interaction network plot. Circles represent the main effect strength of each feature, with larger circles indicating stronger effects. The connections between features represent the interaction strength, with thicker lines indicating stronger interactions between variables. The color gradient reflects the magnitude of both the main effect and interaction strength.

Model performance across different decision thresholds is summarized in Table 3. As the decision threshold increased from 0.05 to 0.50, sensitivity decreased from 100.0 to 52.8%, while specificity increased from 12.4 to 85.4%. At a threshold of 0.20, the model achieved a sensitivity of 92.4% and a specificity of 53.7%, with a number needed to screen (NNS) of 3.4 individuals per PHD case identified. At lower decision thresholds, the model showed higher sensitivity and a lower false-negative rate, but this was accompanied by a higher false-positive rate. As the threshold rises, sensitivity declines while specificity and positive predictive value (PPV) increase, accompanied by a marked reduction in screening rate (Figures 3B,C).

**Table 3:** Model performance at different decision thresholds.

Threshold	Screened (N)	Screen rate (%)	Sensitivity (%)	Specificity (%)	PPV (%)	NPV (%)	NNS
0.05	744	89.7	100.0	12.4	19.4	100.0	5.2
0.08	678	81.8	99.3	21.9	21.1	99.3	4.7
0.10	636	76.7	99.3	28.0	22.5	99.5	4.4
0.12	594	71.7	96.5	33.6	23.4	97.9	4.3
0.15	529	63.8	96.5	43.1	26.3	98.3	3.8
0.18	485	58.5	93.8	48.9	27.8	97.4	3.6
0.20	450	54.3	92.4	53.7	29.6	97.1	3.4
0.25	399	48.1	86.1	59.9	31.1	95.3	3.2
0.30	351	42.3	81.9	66.0	33.6	94.6	3.0
0.35	305	36.8	75.7	71.4	35.7	93.3	2.8
0.40	258	31.1	68.1	76.6	38.0	91.9	2.6
0.45	216	26.1	59.0	80.9	39.4	90.4	2.5
0.50	176	21.2	52.8	85.4	43.2	89.6	2.3

PPV, positive predictive value; NNS, Number Needed to Screen, is defined as the number needed to screen to obtain one positive predictive result, calculated as NNS = 1 / PPV.

## 4 Discussion

This study presents an interpretable, parsimonious machine learning framework for PHD risk screening and stratification in pneumoconiosis. LR demonstrated the most consistent discriminative performance and was selected as the preferred model. Despite the relatively low prevalence of PHD in the external validation cohort (17.4%), the model maintained a stable AUC, suggesting consistent discrimination in the evaluated cohorts. However, the calibrated BSS of 0.173 suggests that while the model outperforms random guessing, its probability estimates remain suboptimal in the external validation cohort. This finding is consistent with the challenges commonly encountered when models developed using retrospective data are applied to contemporary prospective populations (26). This performance gap highlights the importance of temporal and population calibration in model generalizability.

SHAP analysis showed that variables important for model predictions were distributed across hematological, nutritional–metabolic, disease severity, and demographic domains. Our findings indicate that both older age and lower BMI are associated with a higher predicted risk of PHD in patients with pneumoconiosis. The observed association between age and predicted PHD risk is consistent with previous evidence linking older age to chronic disease outcomes (27). A 7-year follow-up study (28) concluded that weight loss for cardiovascular risk prevention should be implemented cautiously and tailored to age, while excessively low BMI may be associated with less favorable cardiovascular outcomes among middle-aged and older adults (29). Pneumoconiosis stage and type reflect differences in disease severity and clinical heterogeneity, and patients with stage III disease and those with coal workers’ pneumoconiosis or silicosis showed higher predicted PHD risk in our model. These findings may partly reflect differences in disease burden and occupational exposure characteristics across pneumoconiosis subtypes (1). A previous study reported higher mortality among patients with coal workers’ pneumoconiosis than among those with other pneumoconiosis types (30), providing broader evidence of differences in clinical outcomes across pneumoconiosis subtypes.

Our study indicates that lower P-LCR, higher MPV, and higher RDW-CV were associated with a higher predicted risk for PHD in patients with pneumoconiosis. Elevated MPV has been associated with platelet activation and chronic inflammatory or hypoxic states in previous studies (31), providing potential clinical context for its contribution to model predictions. RDW has also been investigated as a potential biomarker in pulmonary arterial hypertension (PAH), with lower levels reported in association with better treatment response and prognosis (32, 33). These findings provide clinical context for the association between RDW-CV and model predictions observed in our study. Consistently, a previous multimodal model also identified total protein and P-LCR as important variables in predicting pneumoconiosis risk (34). In addition, lower albumin and higher blood glucose in our study were associated with higher predicted PHD risk, consistent with previous reports linking these variables to nutritional and metabolic status (35, 36). Importantly, the observed SHAP contributions represent statistical associations with model predictions and should not be interpreted as evidence of causal relationships or specific pathophysiological mechanisms underlying PHD.

Although non-smoking status exhibited a slight positive marginal contribution to the SHAP summary plot, this pattern should not be interpreted as evidence of a clinically meaningful protective effect associated with smoking status. The weak main effect and minimal interactions observed for smoking history in the SHAP interaction network suggest that smoking history made a relatively limited contribution to model predictions. This finding highlights the importance of interpreting SHAP values in the context of multivariable relationships and correlated features rather than in isolation (36, 37). Furthermore, the SHAP interaction network revealed prominent statistical interactions among P-LCR, BMI, GLU, and pneumoconiosis stage. These patterns are hypothesis-generating rather than mechanistically definitive and may help characterize combinations of features associated with higher predicted PHD risk, such as advanced pneumoconiosis stage combined with lower BMI and altered blood glucose levels. Beyond improving model interpretability, the interaction network may help generate hypotheses for future studies examining the clinical relevance of these feature combinations.

Beyond model discrimination, this study provides practical information regarding potential clinical implementation through decision threshold analysis. Based on the threshold analysis (Table 3), we propose a three -tier risk stratification framework for clinical decision-making. Patients with predicted probabilities below 0.08 (sensitivity: 99.3%; NPV: 99.3%) may be classified as low risk and managed with routine follow-up, without immediate echocardiographic assessment. Patients with predicted probabilities between 0.08 and 0.20 (sensitivity, 92.4–99.3%) may be considered at intermediate risk and could receive more frequent follow-up and symptom monitoring, with echocardiographic assessment considered if clinically indicated. Patients with predicted probabilities ≥0.20 (PPV, 29.6–43.2%) may be classified as high risk and considered for further clinical assessment, including echocardiography and specialist consultation, as appropriate. This pragmatic risk-stratification framework may help prioritize individuals for further assessment in primary-care and occupational health settings where access to echocardiography may be limited.

This study has several methodological and potential clinical strengths in developing a PHD risk-screening and stratification model for patients with pneumoconiosis. Firstly, based on large-scale Chinese occupational cohorts, we constructed and externally validated an interpretable model. Secondly, the SHAP framework provided both global and individual-level interpretations of model predictions and highlighted statistical interaction patterns among hematological, metabolic, and disease-severity features. Furthermore, decision threshold analysis provided threshold-specific information to support risk stratification and the potential clinical application of the model, particularly in settings where access to advanced assessments may be limited.

This study has several limitations. First, the single-center design and regional variations in pneumoconiosis profiles may limit the generalizability of the findings. Additionally, the EPV of 13.1 in the prospective cohort, although exceeding the conventional minimum of 10, remains relatively modest and may affect coefficient stability. These considerations collectively highlight the need for multi-center external validation in larger cohorts. Second, certain variables (e.g., pulmonary function, CT imaging, and echocardiography) were excluded from the model due to data availability constraints, which may have limited the predictive information available to the model. Nevertheless, patients with pneumoconiosis are often followed in primary-care or occupational health settings, where access to specialized assessments may be limited in routine practice. Therefore, a risk-screening model based on routinely collected clinical and laboratory variables may provide a pragmatic and sustainable approach for early risk stratification of PHD in resource-constrained settings. Finally, although SHAP provides model explanations, it cannot fully replace in-depth research on phenotypes and biological mechanisms, and certain feature directions still require further mechanistic validation. Future studies will involve multicenter, large-scale prospective validation, and multimodal prediction models integrating clinical data, imaging, and biomarkers will be explored to enhance prediction accuracy.

## 5 Conclusion

We developed and externally validated machine learning models for PHD risk screening and stratification among patients with pneumoconiosis using a dual-cohort design. LR achieved the most consistent performance (external AUC = 0.816, BSS = 0.173). SHAP analysis identified clinically accessible variables important for model predictions, including RDW-CV, MPV, albumin, BMI, and pneumoconiosis stage. These findings support the potential utility of machine learning for PHD risk stratification, although further model refinement and prospective validation are needed before clinical implementation.

## Data Availability

The data analyzed in this study is subject to the following licenses/restrictions: data are available on reasonable request. Requests to access these datasets should be directed to Qin Zhang, zhang_q@scu.edu.cn.
