# Supplementary material for: Development and validation of an interpretable machine learning model for pulmonary heart disease in patients with pneumoconiosis
Source: Front Public Health. 2026 Sep 23;14:1952855. doi: 10.3389/fpubh.2026.1952855 (PMC13642772; doi:10.3389/fpubh.2026.1952855)
Supplement: Supplementary file 1 [file Supplementary_file_1.DOCX]

**Supplementary Material for: Development and Validation of an Interpretable Machine Learning Model for the Pulmonary Heart Disease in Patients with Pneumoconiosis**

[Supplementary Table S1 Baseline data of patients with pneumoconiosis complicated with pulmonary heart disease or not in retrospective cohort.	2](#_Toc3849)

[Supplementary Table S2 Brier score and BSS before and after recalibration in internal validation and external validation	3](#_Toc30373)

[Supplementary Table S3 SHAP value of important features.	3](#_Toc10984)

[Supplementary Figure 1 Calibration plots for before and after recalibration in the internal validation cohort t (A, B) and external validation cohort (C, D).	4](#_Toc2763)

| **Supplementary Table S1** Baseline data of patients with pneumoconiosis complicated with pulmonary heart disease or not in retrospective cohort. | | | |
| --- | --- | --- | --- |
| **Feature** | **Pneumoconiosis without PHD**  **(n=802)** | **Pneumoconiosis with PHD**  **(n=182)** | ***P* value** |
| **Age, y** | 52.4±10.4 | 58.1±12.4 | <0.001 |
| **BMI, kg/m²** | 22.8±3.5 | 20.0±3.2 | <0.001 |
| **Dust exposure time, y** | 10.1±7.7 | 13.4±9.8 | <0.001 |
| **Smoker, n (%)** | | | 0.083 |
| yes | 684 (85.3) | 154 (84.6) |  |
| no | 118 (14.7) | 28 (15.4) |  |
| **Pneumoconiosis Type, n (%)** | | | 0.513 |
| Silicosis | 711 (88.7) | 163 (89.6) |  |
| Coal worker's pneumoconiosis | 70 (8.7) | 16 (8.8) |  |
| Other pneumoconioses | 21 (2.6) | 3 (1.6) |  |
| **Pneumoconiosis Stage, n (%)** | | | <0.001 |
| stage I | 209 (26.1) | 22 (12.1) |  |
| stage II | 199 (24.8) | 25 (13.7) |  |
| stage III | 394 (49.1) | 135 (74.2) |  |
| **RDW-SD, fL** | 43.7±4.3 | 46.3±5.2 | <0.001 |
| **RDW-CV, %** | 13.6±1.2 | 14.6±1.9 | <0.001 |
| **WBC, 10⁹/L** | 7.3±3.1 | 9.1±5.1 | <0.001 |
| **MPV, fL** | 10.8±1.6 | 10.3±1.3 | <0.001 |
| **PCT, %** | 0.2±0.1 | 0.2±0.1 | 0.532 |
| **PDW, %** | 15.6±2.0 | 15.3±2.1 | 0.031 |
| **RBC, 10¹²/L** | 4.8±0.6 | 4.8±0.7 | 0.085 |
| **P-LCR, %** | 31.5±11.4 | 28.4±8.9 | 0.002 |
| **TG, mmol/L** | 1.4±0.9 | 1.1±0.6 | <0.001 |
| **TC, mmol/L** | 4.3±0.9 | 4.4±1.1 | 0.741 |
| **LDL, mmol/L** | 2.9±0.8 | 2.9±0.9 | 0.639 |
| **HDL, mmol/L** | 1.2±0.3 | 1.3±0.4 | 0.053 |
| **ALB, g/L** | 38.6±5.2 | 34.6±5.5 | <0.001 |
| **GLU, mmol/L** | 5.2±1.3 | 6.1±2.9 | <0.001 |
| **UA, μmol/L** | 343.3±102.8 | 341.6±115.1 | 0.792 |

| **Supplementary Table S2** Brier score and BSS before and after recalibration in internal validation and external validation | | | | | |
| --- | --- | --- | --- | --- | --- |
| **Cohort** | **Model** | **Raw Brier** | **Calibrated Brier** | **Raw BSS** | **Calibrated BSS** |
| **Internal** | XGB | 0.1340 | 0.1357 | +0.1032 | +0.0921 |
|  | SVM | 0.1262 | 0.1259 | +0.1558 | +0.1579 |
|  | NB | 0.1645 | 0.1334 | -0.1005 | +0.1073 |
|  | LR | 0.1830 | 0.1242 | -0.2241 | +0.1690 |
|  | RF | 0.1377 | 0.1233 | +0.0789 | +0.1748 |
| **External** | XGB | 0.1256 | 0.1376 | +0.1250 | +0.0416 |
|  | SVM | 0.1254 | 0.1280 | +0.1262 | +0.1083 |
|  | NB | 0.1475 | 0.1297 | -0.0276 | +0.0965 |
|  | LR | 0.1398 | 0.1187 | +0.0256 | +0.1727 |
|  | RF | 0.1181 | 0.1279 | +0.1775 | +0.1091 |
| Note:BSS = 1 − (Brier / Brier_baseline). Baseline Brier was 0.1495 for the internal cohort and 0.1435 for the external cohort.Positive BSS indicates improvement over random guessing. | | | | | |

| **Supplementary Table S3** SHAP value of important features. | | | |
| --- | --- | --- | --- |
| **Rank** | **Feature** | **Mean_SHAP** | **Mean_abs_SHAP** |
| 1 | P-LCR | -0.010 | 0.096 |
| 2 | BMI | -0.017 | 0.082 |
| 3 | ALB | -0.069 | 0.077 |
| 4 | Pneumoconiosis Stage | -0.014 | 0.071 |
| 5 | RDW-CV | -0.016 | 0.064 |
| 6 | MPV | 0.001 | 0.061 |
| 7 | Age | 0.025 | 0.055 |
| 8. | GLU | 0.021 | 0.049 |
| 9 | Smoker | 0.014 | 0.018 |
| 10 | Other Pneumoconiosis Type | -0.006 | 0.009 |

**Supplementary Figure 1** Calibration plots for before and after recalibration in the internal validation cohort t (A, B) and external validation cohort (C, D).
